# Examining online international health professions education: a mixed methods review of barriers, facilitators, and early outcomes★

**DOI:** 10.1051/ject/2023044

**Published:** 2024-03-15

**Authors:** Laura Dell’Aiera, David Fitzgerald, David Fisher, Norman W. Gill

**Affiliations:** 1 Medical University of South Carolina, Division of Cardiovascular Perfusion Charleston SC 29425 United States; 2 George Washington University, School of Medicine and Health Sciences, Department of Health Human Function and Rehabilitation Washington DC 20052 United States; 3 Medical University of South Carolina Hospital Authority, Department of Cardiovascular Perfusion Charleston SC 29425 United States

**Keywords:** Health professions, International, Education, Perfusionist

## Abstract

*Background*: Access to quality healthcare education across the world is disproportionate. This study explores the potential for Cardiovascular Perfusion education to be delivered online to reach international students. *Methods*: Exploratory mixed methods were used to identify the barriers, facilitators, and early outcomes of online international health professions education. *Results*: Qualitative analysis yielded four primary and nine subthemes. Multiple interventions were implemented in the planning of a novel online international Extracorporeal Science (ECS) program based on these themes. Quantitative data from the first semester of the new ECS program was collected along with data from the traditional entry-level program and historic data from previous entry-level cohorts. No significant correlations or differences were found between students. Student satisfaction surveys were determined to be equivalent for each group. Mixed data analysis revealed exceptional student satisfaction in areas where qualitative feedback was incorporated into the program design. *Conclusions*: Online international education may be a viable option in the health professions. Barriers and facilitators to this mode of education were identified and utilized in designing one such program. Early outcomes from the novel ECS program reveal that student performance and satisfaction are equivalent to those of a traditional in-person training program.

## Introduction

Public health research has estimated that 93% of those in need of cardiac care in developing nations do not have access and will go untreated [1]. The challenges to healthcare access are multifactorial but one contributor is a lack of trained personnel [2]. The field of Cardiovascular Perfusion (CVP) is a specialty within cardiac surgery whose professionals operate the heart-lung bypass machine (HLM) to make surgical operation on the heart possible. In the nearly 70 years since its first use, HLM technology has advanced and the field of CVP has increased in complexity necessitating more robust training, education, and knowledge from the clinicians in this field [3, 4].

Although it is difficult to accurately estimate the number of perfusionists worldwide or the actual need for new graduate perfusionists, the literature suggests that the availability of academic perfusion training across the globe is limited. A literature search revealed that only 19 of the 194 countries in the world have perfusion education programs or published literature. Further research shows that the availability of trained professionals, medical resources, and access to cardiac care globally are highly disproportionate, irrespective of population [2].

In a review of perfusion education programs, Stammers identified the primary reason for program closure was due to financial viability [5]. With the niche nature of the field and the very small number of trainees, the financial impact is even greater in small programs or low-income nations. Tuition is the major source of income for academic programs [6] and with very few students, costs cannot be balanced [5, 7]. Universities are unable to support perfusion education programs in regions where professional vacancies are infrequent and therefore class sizes are very small due to limited demand. For these reasons, the primary mechanism of perfusion training in many countries around the world is on-the-job training.

While on-the-job training may provide the clinical and technical skills that a perfusionist requires, didactic education and theoretical foundation are not available to clinicians trained in this manner. In some regions “self-study” materials are provided to trainees for independent exploration [7, 8]. In other regions, no such academic guidance is provided. As research in medicine has shown, with increased levels of education, patient outcomes also increase [9].

It is for these reasons that the pursuit of an alternative educational strategy is necessary. At the (*Medical University of South Carolina* (*MUSC*)) a novel Extracorporeal Science (ECS) program was proposed to educate perfusionists around the world. The program faculty saw the need expressed through international colleagues and an opportunity to take their resident curriculum to a broader audience. The program is structured to deliver the didactic education of a traditional Perfusion Education program with the expectation that it will supplement the on-the-job clinical training that students will receive in their global regions. This program was structured to be delivered in English and English fluency was a prerequisite for entry. All courses were taught asynchronously and remotely by the Cardiovascular Perfusion faculty at (*MUSC*) in the United States.

As the first to develop such a program, we investigated the challenges that may be involved. This research study sought to determine how identified barriers and facilitators to online international health professions education impact the effectiveness of a novel Cardiovascular Perfusion education program.

## Materials and methods

This study involved exploratory, sequential mixed methods research in which the qualitative phase preceded the quantitative. This style of mixed methods research is appropriate for topics that have little or no previous research data available [10]. The authors approached each research phase with separate paradigms. The qualitative phase was approached through a constructivist framework to understand the experiences of interviewees. The quantitative phase was approached through a post-positivist framework to objectively collect outcomes data.

The topic of online international health professions education has little to no published literature. For this reason, an open exploration to understand the experiences of health professions educators who have taught international or online students was pursued. Institutional review board (IRB) approval was received from the (*MUSC*) IRB prior to recruitment and interviews (Pro00118039). Semi-structured interviews were conducted over video conferencing technology with electronic recordings and transcripts being gathered after verbal consent from interviewees.

Transcripts were deidentified and subjects were given identification (ID) numbers by the primary investigator (PI) who conducted all the interviews to maintain consistency. Interviews continued until saturation was achieved and no new information was discovered. Deidentified data was shared with a co-investigator and both PI and co-investigator used thematic analysis to independently analyze the interviews for recurring or profound themes. Triangulation was used to compile the identified themes and investigators continued review until consensus on relevant themes was achieved.

The quantitative analysis centered on the Resident 2022, Online 2022, and Resident 2021 cohorts. The Resident 2021 cohort received content in the traditional in-person lecture-based format in (*Charleston*, *South Carolina at MUSC*). The Resident 2022 and Online 2022 cohorts participated in a hybrid format with a flipped classroom style [11] of content delivery which introduces pre-class teaching materials for independent review and reserves classroom time for discussion surrounding the application of the material [12]. Resident 2022 students were physically present for the conversation and Online 2022 students viewed the recorded activities and participated asynchronously where applicable.

Course outcomes were measured through course grades as well as academic progress from a baseline written examination to the final written examination in two separate courses each with a separate instructor. Instructors were consistent for all three student cohorts. Student satisfaction was measured by a university-administered survey which included eight Likert-scale questions rated from 1 to 5 on an agree/disagree scale (Table 1). All data were checked for errors and outliers. Descriptive statistics were run for all variables, including assumptions for parametric testing. The alpha level was set at 0.05 for all tests of statistical significance and analysis was completed in SPSS software, version 28 (Armonk NY, IBM).

**Table 1:** Student satisfaction survey.

Questions	
Q1	Learning objectives were clearly defined
Q2	The syllabus adequately explained how assignments were graded and how course grades were earned.
Q3	Course activities contributed significantly to learning the course material.
Q4	The educator encouraged appreciation of other healthcare professionals.
Q5	The educator was an effective teacher.
Q6	The educator was well prepared.
Q7	The educator was responsive to students’ questions and concerns.
Q8	The educator facilitated an interactive learning environment.

Student satisfaction evaluated by Likert-scale from 1 = strongly disagree to 5 = strongly agree.

Statistical analysis compared the two Resident cohorts to determine if the change in curriculum and delivery impacted course outcomes. A comparison of final course grades between the 2021 and 2022 Resident cohorts was performed using independent *t*-tests. Then, a comparison was done between the 2022 cohorts to determine if resident versus non-resident status impacted course outcomes. Due to the imbalance in cohort sizes, a Mann–Whitney *U* test was used to compare final course grades between these two groups.

The qualitative interviews with experienced educators had suggested associations between demographic data and course outcomes, so the correlational analysis was used to examine this possibility. Associations included gender to course grade (Point Biserial); age to course grade (Pearson’s *r*); years in healthcare to course grade (Pearson’s *r*); and highest degree earned prior to matriculation to course grade (Spearman’s Rho).

Differences between the baseline and final written examinations were investigated using a 2 × 2 mixed model ANOVA. The two-time points were the baseline written examination and final written examination, and the two groups were the Online 2022 and the Resident 2022 cohorts.

Finally, student satisfaction surveys from all three cohorts were compared and analyzed to determine trends in student satisfaction. Mann–Whitney *U* tests were utilized to make this comparison.

The final step in the analysis was to strengthen the findings by combining the qualitative and quantitative results. The themes that were identified during the qualitative interviews were merged with the quantitative outcomes and survey questions to identify if the discussed barriers and facilitators were reflected in the curriculum implementation. These analyses helped to determine program effectiveness and if program changes adequately accounted for, or capitalized on, the identified themes.

## Results

Cohort demographics are described in Table 2. Although open to all international students, the initial Online cohort was recruited from the regions of Australia and New Zealand. There were more females enrolled in both the Online 2022 and Resident 2022 cohorts, while the Resident 2021 cohort included slightly more males. The average age was similar between groups. Years of previous healthcare experience were lowest in the Resident 2021 cohort at a mean ± SD of 2.7 ± 4.7 years and highest for the Online 2022 cohort at 4.5 ± 5.7 years. Finally, there was one student in each of the Online 2022 and Resident 2021 cohorts with a graduate degree prior to matriculation. The Resident 2022 cohort included four students with previous graduate degrees.

**Table 2:** Demographics.

Characteristics	Resident 2021 (*N* = 28)	Resident 2022 (*N* = 29)	Online 2022 (*N* = 5)
Male-no. (%)	16 (57.1)	9 (31.0)	1 (20.0)
Age-years ± SD	27.9 ± 5.2	26.2 ± 4.4	28.2 ± 4.4
Healthcare experience-years ± SD	2.7 ± 4.7	3.0 ± 3.3	4.5 ± 5.7
Graduate degree-no. (%)	1 (3.6)	4 (13.8)	1 (20)

SD: Standard Deviation.

### Qualitative results

Saturation was achieved with 17 interviews after which triangulation ensued. Four primary and nine subthemes were agreed upon and can be found in Table 3.

**Table 3:** Qualitative themes.

Themes	Codes	Description	Educator quotes
*Culture*	CUL	Differences in culture between student, instructor, and peers may impact barriers and facilitators.	“In Kenya, if you say, “we’re meeting at 6:00.” You’re on time until 6:59.”
Mutual Learning	MUT	Learning happens for domestic and international students as well as for the educator due to the diversity in the cohort.	“I get to learn a lot about people from different cultures and I think that makes me a better teacher.”
System Differences	SD	Differences in healthcare systems and educational systems will impact the process and product.	“It’s such a different educational model… They get government sponsors… have to work as a trainee for two years and they go to class one day a week.”
*Time Commitment*	TIME	Educating online, asynchronously, across time zones requires more time from the educator and the student than a traditional in-person, domestic program.	“Commit to it like it were a class. But… that can’t be 2 h on Tuesday and Thursday. It needs to be 30 min three times a day, 5 days a week.”
Preparation	PREP	Educators and students must be prepared and organized.	“This isn’t something that you can just say ‘oh we’re going to become an online program now’”
Boundaries and Flexibility	B&F	Differing experiences on the expectations of instructors to be available to students.	“Students, when they randomly post something in the middle of the night, expect to have an answer at breakfast in the morning.”
*Relationship*	REL	Relationships are critical for student success. While more challenging to build in the online format, it is possible.	“Relationship building is absolutely more difficult [but] not impossible. You have to work for it a lot harder.”
Selecting Participants	SEL	Certain selection criteria may contribute as a facilitator or barrier to success.	“We’ve suggested that they have some experience working in a hospital and with patients.”
Student Characteristics	STU	Successful students tend to display certain skills and exhibit certain characteristics.	“To be successful in the program requires a constellation of traits.”
Faculty Characteristics	FAC	Effective educators tend to display certain skills and exhibit certain characteristics.	“They [faculty] really need to be passionate about this stuff.”
*Practical Considerations*	PRA	Experts emphasized certain practical considerations to program organization for optimal results.	“Time difference on multiple levels is a challenge.”
Language	LAN	International education presents the practical challenge of disparate language.	“One of the greatest challenges is the language barrier.”
Access	ACC	Access in terms of students, educators, and geography.	“You can invite an expert… on a particular topic… into the [virtual] classroom.” “You can bring these thought leaders right to these people.”

Identified themes from educator interviews. Primary themes are shaded, subthemes are in white.

#### Culture

Several educators emphasized the impact of culture on the learning environment with international cohorts. Differences in cultural upbringing and respect were seen to impact the exchange of conversation and knowledge within the classroom. Some educators noticed that “respect cultures”, prevalent in certain regions of the world, cause students to hesitate to ask questions or admit confusion for fear of disrespecting the overseeing educator. Representative quotes include:

*“Culturally they’re very respectful…nobody hardly ever questions me in those countries.”*

*“‘I’m a fallible human being… but you have to keep reinforcing that… “we were stronger because you didn’t defer to my age or experience… we did this together”.”*

However, once educators felt that there was sufficient trust and exchange of information, the opportunity for mutual learning from educator to student, student to student, and student to educator was enhanced by the diverse group. Many educators recounted specific examples of times that they gained new knowledge while teaching an international cohort of students.

#### Time

Another recurring theme was the time commitment that is required to educate students online and internationally. Several educators mentioned that more preparation than what is common in traditional classrooms is necessary to successfully conduct an online international program. For example:

*“…it took us three years to… get it all created and recorded… We go to a studio. They have people that do makeup. They have a whole setup where they have you record your lectures. It’s very professionally done.”*

With students in various time zones and a variety of life and work dynamics, the impact on educators can be intrusive to normal working hours which caused several educators to emphasize the importance of setting boundaries and recognizing when flexibility was necessary.

#### Relationships

Relationships were important to educators when considering the experience for students and encouraging retention within the program. Educators felt that relationship building was more difficult online but could be enhanced with purposeful communication through advisor-advisee pairings and occasional synchronous meetings for personal connection. One educator noted:

*“It actually helps us give honest feedback because we know the students so well.”*

Finally, several characteristics of both students and educators were believed to contribute to facilitating success with online international education.

#### Practical considerations

Several practical considerations were identified as critical to identifying barriers and facilitators to online international education. Amongst the recurring topics were time zones and languages. Time zones were considered a manageable barrier with the primary suggestion being the establishment of a programmatic operating time zone. Language barriers require further consideration and exploration. In many countries, English is considered the language of medicine. However, many educators emphasized the need to ensure students fully understand the technical terminology, particularly when it is taught in a non-native language. Educators shared that it is important to avoid idioms, slang, and jokes that may not translate to other languages. One educator said:

*“My students do speak English but their comprehension in English is not going to be as good as their comprehension in their native tongue.”*

Other practical considerations included access in various forms. For students, the flexibility of online asynchronous learning allows them to continue working and mitigate the financial strain of returning to school. Additionally, giving education to the student and allowing them to remain in their region will encourage them to practice in that area, dissuading the “brain drain” phenomenon [13]. However, access in certain regions of the world to reliable connectivity and technology at a cost that students can afford may still be of concern. For educators and universities, this educational model improves the ability to integrate work and life and improves access to expert educators across the globe as opposed to being restricted to a particular geographic region. One educator had this experience to share:

*“You can invite an expert… on a particular topic… into the [virtual] classroom... It’s amazing- the technology. You can bring these thought leaders right to these people.”*

### Quantitative results

Final course grades were compared between the three groups for two separate courses, CVP-700 and CVP-702. Independent *t*-tests used to compare the Resident 2021 and 2022 cohorts revealed no significant difference (*t* = 0.45, *p* = 0.66). Likewise, Mann–Whitney *U* tests between the Resident 2022 and Online 2022 cohorts revealed no significant difference (*U* = 50.5, *p* = 0.29).

Demographic data and course grade associations were investigated. No significant correlations were found to exist between gender, age, years of healthcare experience, or highest degree earned and final course grade. Correlations data can be found in Table 4.

**Table 4:** Correlations.

Correlations to course grade	Resident 2021 CVP-700	Resident 2021 CVP-702	Resident 2022 CVP-700	Resident 2022 CVP-702	Online 2022 CVP-700	Online 2022 CVP-702
Gender	*P* = 0.475	*P* = 0.517	*P* = 0.709	*P* = 0.175	*P* = 0.575	*P* = 0.752
Age	*P* = 0.318	*P* = 0.414	*P* = 0.292	*P* = 0.745	*P* = 0.828	*P* = 0.956
Years in healthcare	*P* = 0.822	*P* = 0.921	*P* = 0.253	*P* = 0.769	*P* = 0.285	*P* = 0.398
Highest degree earned	*P* = 0.161	*P* = 0.161	*P* = 0.115	*P* = 0.320	*P* = 0.182	*P* = 0.559

CVP-700: course code; CVP-702: course code.

ANOVA comparison of the pre and post-examination scores between the Resident 2022 and Online 2022 cohorts revealed no interaction effect for CVP-700 (*F* = 2.43, *p* = 0.13). The main effects were then examined and found to be significant for both time (*F* = 172.38, *p* = <0.001) and cohort (*F* = 6.13, *p* = 0.02). For CVP-702, there was a significant ordinal interaction effect (*F* = 4.17, *p* = 0.05). Therefore, we interpreted the significant main effects for both time (*F* = 80.42, *p* = <0.001), with the post-test always being higher, and cohort (*F* = 11.66, *p* = 0.002) with the Online cohort always being higher.

**Figure 1:**
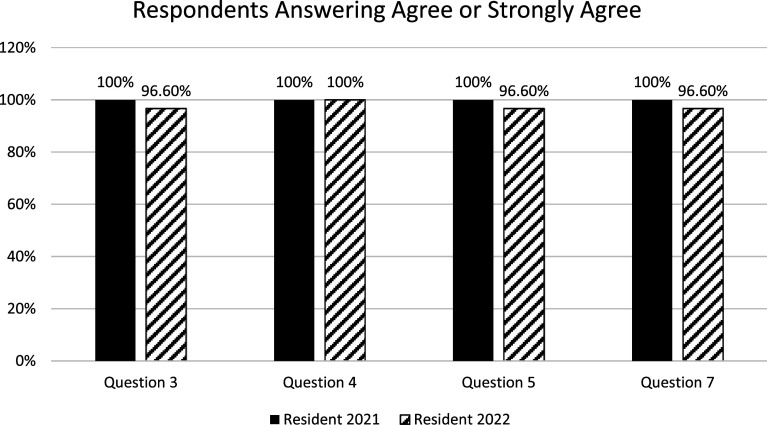
Satisfaction survey results. Legend: Questions with statistical significance were analyzed for positive responses. Positive responses are reported as a percent of total responses.

Student satisfaction as measured through Likert-scale questions revealed statistically significant differences for questions 3 (*p* = 0.48), 4 (*p* = 0.032), 5 (*p* = 0.029)*,* and 7 (*p* = 0.026) between the Resident 2021 and Resident 2022 cohorts. Descriptive statistics were analyzed to determine the relevance of this difference. Median and mode satisfaction scores for these questions were 5.0 for both groups, suggesting there is no practical difference in satisfaction scores. Additionally, if the agree and strongly agree categories are collapsed together to indicate “satisfaction” then 96% and 100% of students were satisfied in the Resident 2022 and Resident 2021 cohorts, respectively (Figure 1). Additionally, there were no significant differences in satisfaction between the Resident 2022 and Online 2022 cohorts.

### Combined results

The qualitative themes, corresponding interventions, and corresponding quantitative outcome data indicators are displayed in Table 5. Each theme was considered independently, and program interventions were designed to account for the barriers and facilitators within the theme. These interventions are available in column 2 of Table 5. Table 6 displays qualitative themes, interventions, and quantitative student satisfaction results. Certain questions from the university-administered student satisfaction survey were identified as being related to the qualitative themes and interventions. The data reported in Tables 5 and 6 show that after the incorporation of the interventions into the program planning, there were no significant differences between cohorts in course outcomes or student satisfaction.

**Table 5:** Qualitative themes and course outcomes.

Theme	Integration into curriculum	Course outcomes	Quantitative finding
CUL	Incorporate international healthcare systems and standards	Pre-post test difference; 2 × 2 ANOVA Final course grade comparisons; independent *t*-test and Mann–Whitney *U*	The ECS cohort consistently scored higher on the pre and post test written examinations. There was significant improvement over time in both cohorts. There was no significant difference between cohorts in final course grades (*p* > 0.05).
TIME	Development of activities that engage online cohorts (flipped classroom)	Pre-post test difference; 2 × 2 ANOVA Final course grade comparisons; independent *t*-test and Mann–Whitney *U*	The ECS cohort consistently scored higher on the pre and post test written examinations. There was significant improvement over time in both cohorts. There was no significant difference between cohorts in final course grades (*p* > 0.05).
PREP	Planning meetings prior to implementation	Pre-post test difference; 2 × 2 ANOVA Final course grade comparisons; independent *t*-test and Mann–Whitney *U*	The ECS cohort consistently scored higher on the pre and post test written examinations. There was significant improvement over time in both cohorts. There was no significant difference between cohorts in final course grades (*p* > 0.05).
B&F	Asynchronous interactions between students and teachers, periodic synchronous group meetings	Pre-post test difference; 2 × 2 ANOVA Final course grade comparisons; independent *t*-test and Mann–Whitney *U*	The ECS cohort consistently scored higher on the pre and post test written examinations. There was significant improvement over time in both cohorts. There was no significant difference between cohorts in final course grades (*p* > 0.05).
REL	Synchronous meetings between assigned advisor-advisee pairs	Pre-post test difference; 2 × 2 ANOVA Final course grade comparisons; independent *t*-test and Mann–Whitney *U*	The ECS cohort consistently scored higher on the pre and post test written examinations. There was significant improvement over time in both cohorts. There was no significant difference between cohorts in final course grades (*p* > 0.05).
SEL	Student selection based on healthcare experience and proven academic record	Correlations; Pearson’s *r*, Spearman’s Rho	No significant correlation between years of healthcare experience or highest degree earned and final course grade was found (*p* > 0.05).
STU	A diverse and representative cohort was sought	Correlations; point biserial; Pearson’s *r*	No significant correlation between gender or age and final course grade was found (*p* > 0.05).
PRA	Set time zone standards (EST), periodic synchronous meetings	Pre-post test difference; 2 × 2 ANOVA Final course grade comparisons; independent *t*-test and Mann–Whitney *U*	The ECS cohort consistently scored higher on the pre and post test written examinations. There was significant improvement over time in both cohorts. There was no significant difference between cohorts in final course grades (*p* > 0.05).

Theme codes, implementations into the program, and quantitative outcome items used as correlate measures. Primary themes are shaded, subthemes are in white. B&F: Boundaries and Flexibility; CUL: Culture; ECS: Extracorporeal Science; PRA: Practical Considerations; PREP: Preparation; REL: Relationship; SEL: Selecting Participants; STU: Student Characteristics.

**Table 6:** Qualitative themes and student satisfaction.

Theme	Integration into curriculum	Survey question	Student satisfaction Resident 2022	Student satisfaction Online 2022
MUT	Incorporate international healthcare systems and standards	Appreciating other professionals (Q4)	100%	100%
TIME	Development of activities that engage online cohorts (flipped classroom)	Course activities (Q3)	96.6%	100%
TIME	Additional assignments designed to engage online learners and test knowledge	Interactive learning (Q8)	100%	80%
PREP	Quality Matters certification	Learning objectives (Q1)	96.6%	100%
PREP	Quality Matters certification	Course syllabus (Q2)	100%	100%
PREP	Planning meetings prior to implementation	Preparation (Q6)	100%	100%
B&F	Asynchronous interactions between students and teachers, periodic synchronous group meetings	Interactive learning (Q8)	100%	80%
REL	Synchronous advisor-advisee meetings	Responsiveness (Q7)	96.6%	100%
PRA	Set time zone standards, periodic synchronous meetings	Responsiveness (Q7)	96.6%	100%

Theme codes, implementations into the program, and student satisfaction items were used as correlate measures. Primary themes are shaded, subthemes are in white. Student satisfaction is percent of respondents that chose an affirmative response. B&F: Boundaries and Flexibility; MUT: Mutual Learning; PRA: Practical Considerations; PREP: Preparation; REL: Relationship.

## Discussion

Distance education is a novel possibility for expansion to areas of the globe and populations without access to advanced education. The literature is sparse concerning online international health professions education and mostly inferred from the adaptation of traditional to online education in response to the COVID-19 pandemic [14]. A systematic review found reports that spanned from overall positive student perception to overall negative student perception with variances across access, course design, and skills attainment [14]. However, none of these articles look at programs designed from the start for the online environment, or educators and learners in different geographies and cultures.

Studies looking at cultural influences in the classroom such as classroom behaviors [15], learning preferences [16, 17], and feedback preferences of students from different cultures [18] are available. However, these publications do not address health professions specifically, and therefore our study helps fill a gap in the literature. We found that educators experienced in relevant areas were able to identify perceived facilitators and barriers to online international education and felt that it was a viable option for expanding access to healthcare education across the globe. The most relevant themes were shared with the Extracorporeal Science (ECS) program faculty.

The recurring themes that were most pertinent to the research question were incorporated into the planning phases of the online ECS program. Several changes were made in the proposed program ranging from practical and organizational to curricular. Specifically, consideration of the theme of preparation led the faculty to pursue Quality Matters certification [19]. This is a nationally recognized certification that provides guidance for formatting and delivery of online learning. Within the same theme, the time, effort, and energy required to maintain student engagement in the online setting was a primary concern. To address this area, the faculty reformatted one of two hybrid courses to a flipped classroom model as several interviewees agreed that it encourages engagement. One participant described the flipped classroom online interaction like this:

*“You’ve learned it. You’ve read the materials, you watched the webcast, you’ve taken the quiz, and you’ve gotten feedback from that. Now let’s dig deeper. Let’s discuss this topic.”*

Mutual Learning was incorporated through efforts to include geographic or culturally relevant information into the curriculum and courses. Practical considerations led to the decision that the designated program time zone would be U.S. Eastern Standard Time (EST) and that there would be periodic synchronous meetings between the faculty and ECS students which would require flexibility from the educator. Finally, faculty advisors were assigned to each ECS student to promote relationship building and synchronous meetings were required at least once per semester.

When considering prospective students for the first ECS cohort, academic records, previous accomplishments, and healthcare experience served as selection criteria and were consistent with those that educators identified as potential indicators for success. Additionally, diversity amongst the ECS group was desired and allowed consideration of gender and age correlations with course grades.

Analysis of early outcomes of the ECS program revealed that incorporating educator experiences in the planning phases of an online international program may be key to successful implementation. Our experience revealed that content delivery and education are possible and can be as effective as traditional in-person education. Specific student characteristics identified by educators as being potential predictors of success were not found to correlate with outcomes as previously indicated (Table 2). Overall, our students were equally satisfied with in-person traditional content delivery, flipped classroom resident content delivery, and flipped classroom online asynchronous content delivery.

While early outcomes from this experience are promising, the data represent one specialty graduate program in one institution with a small cohort. Additionally, this report includes only one semester’s worth of course grade outcome data.

Other limitations within the study are related to Likert-scale measurements. Analysis and interpretation of Likert-scale data are challenging when determining a practically significant difference in ratings. Additionally, the pre-constructed university-templated questions limited specificity to the unique themes revealed in the qualitative phase.

Finally, the researchers elected to remove an outlier data point that was five standard deviations above the mean and determined to be non-representative of the group. A sensitivity analysis demonstrated that the removal of this data point impacted the correlation between age and course grade, changing the result from significant to non-significant.

## Conclusion

Ultimately, the potential implications of success in this novel program could be of benefit to the health professions education community. Expanding health professions education through online international programs could positively impact the availability of high-quality didactic curriculum, and knowledgeable clinicians and educators, to areas of the world where this is currently not feasible. To strengthen these findings, it will be important to track and report the long-term outcomes of students and graduates from the ECS cohort as well as continued research into other indicators of success.

(https://chp.musc.edu/academics/cvp/extracorporeal-science)

## Data Availability

The interview guide used in this study is available in Zenodo in [20]. The research data are available on request from the authors.
